# The effectiveness of a problem-solving intervention with workplace involvement on self-reported sick leave, psychological symptoms and work ability: a cluster randomised clinical trial

**DOI:** 10.1186/s12889-024-20564-z

**Published:** 2024-11-05

**Authors:** Andreas Eklund, Ida Karlsson, Gunnar Bergström, Holmlund Lisa, Björk Brämberg Elisabeth

**Affiliations:** 1 Institute of Environmental Medicine, Unit of Intervention and Implementation Research for Worker Health, Karolinska Institutet, Karolinska Institutet, 171 77, Stockholm, Box 210, Sweden; 2 Department of Occupational Health, Psychology and Sports Science, Faculty of Health and Occupational Studies, University of Gävle, Gävle, Sweden; 3 Department of Neurobiology, Care Sciences and Society, Division of Occupational Therapy, Karolinska Institutet, Stockholm, Sweden; 4 School of Public Health and Community Medicine, Institute of Medicine, University of Gothenburg, Gothenburg, Sweden

**Keywords:** Common mental disorders, Depression, Anxiety, Adjustment disorder, Problem-solving, Workplace involvement, Primary care, Cluster-randomised trial, Sick leave

## Abstract

**Background:**

Problem-solving interventions with workplace involvement (PSI-WPI) have been shown to reduce sick leave and increase return to work in an occupational health services context. However, many employees struggle with reduced work functioning, anxiety-, and depressive symptoms up to 12 months after a sick leave episode, and it is unclear if the intervention affects outcomes other than sick leave. The aim of this study is to investigate if a PSI-WPI added to care as usual (CAU) is superior to CAU with respect to self-reported sick leave, psychological symptoms, work ability, work performance, and health after RTW when provided in primary care.

**Methods:**

Employed individuals aged 18–59 years on sick leave (2 to 12 weeks) diagnosed by a physician at a primary care center with mild to moderate depression, anxiety, or adjustment disorder were enrolled in a two-armed cluster-randomised trial evaluating the effectiveness of a PSI-WPI. Multiple outcomes were recorded at baseline, six months, 12 months, and every fourth week during the study period. Outcomes were categorised into psychological symptoms, health, work ability, work performance, and self-reported sick leave. Data were analysed using MANOVA, GEE (Generalized Estimating Equations), and cox regression.

**Results:**

One hundred ninety-nine individuals responded to the invitation to participate; one participant withdrew, one was excluded as the employment ended, nine did not answer the baseline survey, and three were removed from the analysis due to missing data. The analysis included 81 subjects who received the intervention and 104 subjects who received the control. Baseline characteristics were similar across both groups. No differences between the groups were found among either variables except one. There was a significant difference between the groups in self-rated health (EQ5D) in favour of the CAU group from baseline to six-month follow-up, with a mean difference of -8.44 (-14.84, -2.04).

**Conclusions:**

A problem-solving intervention with workplace involvement added to CAU did not result in statistically significant reductions in outcomes. Further research is needed to understand why problem-solving interventions appear to have an effect on sick leave in an occupational health services context and not in a primary care context.

**Trial registration:**

NCT3346395, registration date 2017–11-17.

**Supplementary Information:**

The online version contains supplementary material available at 10.1186/s12889-024-20564-z.

## Introduction

Approximately one in five employees are affected by common mental disorders (CMDs) (i.e., mild- to moderate depression, anxiety, adjustment, and stress-related disorders) at any given time, and lifetime prevalence reaches 30% to 50% depending on which diagnoses/disorders are studied [1, 2]. The burden of poor mental health is substantial worldwide. Among the Organization for Economic Cooperation and Development (OECD) countries, the economic costs of CMDs have been estimated to be more than 4.2% of the gross domestic product, where more than a third of this is due to lower employment rates and lower productivity at work [3, 4]. As a result, individuals and families report significant health consequences and reduced abilities to contribute to their communities [5]. In Sweden, CMDs are the most common cause of sick leave, representing almost half of all ongoing sick leave spells [6]. Moreover, employees with CMDs have longer sick leave periods and have an increased risk of recurrence compared to employees on sick leave for other diagnoses [7].

Understanding sick leave for employees with CMDs is complex, and it is of interest to identify the mechanisms involved in the return-to-work (RTW) process [8, 9]. Employees with CMDs appear to benefit from being employed, especially if work conditions are favourable and high-quality supervision is available [10]. Nevertheless, there are also risk factors for CMDs in the work environment, such as high workload, low control (job strain), low reward, poor social support at the workplace, and lack of fairness at the workplace [11, 12]. In addition, these work environment risk factors have been associated with cardiovascular disease, musculoskeletal disorders, and sick leave [13–18]. Moreover, due to the complex factors behind sick leave due to CMDs [19–21], psychological symptoms and work ability outcomes beyond sick leave are relevant to consider in intervention research [22].

The intervention evaluated in the present study, hereafter named PSI-WPI, is a problem-solving intervention with workplace involvement in primary care in Sweden. PSI-WPI is a five-step problem-solving process that aims to identify problems and solutions for RTW and has been found effective in reducing sick leave days in an occupational health services (OHS) context [8, 23, 24]. In line with the intervention evaluated in the present study, effective interventions for employees on sick leave due to CMDs usually include a clinical intervention (e.g., cognitive behavioral therapy or problem-solving) and a workplace intervention (e.g., involvement of the manager) [25–29]. Previous studies show that combining a problem-solving intervention and workplace involvement can reduce the number of sick leave days and time to partial RTW during the first year and the time to first RTW [27, 28].

The PSI-WPI primarily aims to reduce the number of registered sick leave days (e.g., sick leave days > 14 days); we may also expect changes in psychological symptoms, work ability, and health. Psychological symptoms such as depression, anxiety, and sleeping problems are important because of the suffering, reduced life quality, and impaired work ability they may contribute to [30–32]. In addition, it is relevant to evaluate self-reported sick leave because it also includes short-term sick leave (≤ 14 days) not registered in Swedish Social Insurance data. Reduced short- and long-term sick leave is beneficial from employer and societal perspectives [33].

Work functioning is also relevant to consider when evaluating the PSI-WPI. For example, a recent RCT evaluated the work functioning of employees who had returned to work after a problem-solving intervention. They found that many still struggled with impaired work performance, anxiety-, and depressive symptoms up to 12 months post sick leave due to a CMD [34]. A systematic review furthermore found that work-focused problem-solving skills decreased days with sick leave and the duration of sick leave, but the interventions did not significantly improve psychological symptoms [27]. Similarly, a systematic review analysing the effect of clinical interventions in combination with a workplace intervention showed an effect on decreased depressive symptoms but found no association with work functioning [25]. More research is needed to understand these associations and establish the potential benefits of problem-solving interventions with workplace involvement in primary care. Furthermore, the effectiveness of PSI-WPI for CMDs in a primary care setting has not been established.

The aim of this study is to investigate if a PSI-WPI added to care as usual (CAU) is superior to CAU. We hypothesize that participants who have undergone the problem-solving intervention will have a reduction in self-reported sick leave, psychological symptoms and an increase in work ability, work performance, and health after RTW compared to the participants who receive CAU, when provided in primary care.

## Methods

The study used baseline and follow-up data from the PROSA trial (Problem-solving intervention in primary care), a two-armed cluster-randomised trial (reg. NCT3346395). Ethical approval was obtained from the regional ethical review board in Gothenburg (reference number 496–17). The reporting for this study follows the CONSORT guidelines. The design and procedures for the RCT are described in a study protocol [8]. A summary of methods for the present study is presented below.

### Participants and recruitment

Inclusion criteria were; employed women and men aged 18–59 years on sick leave (i.e., a minimum of 2 weeks and a maximum of 12 weeks) diagnosed with mild to moderate depression, anxiety, or adjustment disorder (F32, F41, F43) as the primary cause; they were diagnosed by a physician at one of the included primary care centers (PCC); accepted the employer’s involvement; and understood written and spoken Swedish. Exclusion criteria were severe depression; other severe mental disorders (i.e., psychotic or bipolar disorders or referral to a psychiatrist); pregnancy; somatic complaints; or disorders that affect workability. Recruitment lasted 24 months, from February 2018 until February 2020. Rehabilitation coordinators (RCs) were recruited from PCCs in the Västra Götaland region, Sweden. The 9 RCs providing PSI-WPI were all female with a mean age of 57 years (range 39–68), and five had worked more than three years as an RC. Their professions were registered occupational therapist, registered physiotherapist, registered nurse, or unknown. Of the 10 RCs providing CAU, eight were female, with a mean age of 52 years (range 33–64), and seven had worked more than three years as an RC. A primary care assistant, blinded for group allocation, recruited employees on sick leave through the screening of medical records. Employees who met the criteria for inclusion received information about the study via email.

### Randomisation and blinding

Randomisation was conducted by means of computer-generated random numbers stratified according to the PCCs care-need index. The index is a compounded sociodemographic variable (e.g., education, single household, or single parent) reported on group level for each PCC and is expected to impact the outcome. Due to the training of RCs in the intervention arm, the RCs were not blinded to treatment allocation. To avoid contamination, all RCs – irrespective of randomisation – received instructions not to share information about the intervention with others nor inform employees of their allocation. The employees received identical information about the study and were blinded to treatment allocation. An independent and blinded statistician performed the analysis.

### The problem-solving intervention with workplace involvement

The PSI-WPI was delivered on top of CAU. RCs in the intervention arm participated in a two-day training session provided by a licensed psychologist and the principal investigator (EBB). The RCs were also supported by a manual and worksheet and had opportunities for supervision and feedback.

The PSI-WPI is based on the SHARP-at-work intervention [35] and builds on a participatory approach but was provided to employees in primary health care instead of occupational health.

care [23]. The modified version consists of five steps aiming to identify problems and solutions that are barriers to RTW. 1) An inventory of problems related to RTW (RC and employee), where after the inventory, the RC contacts the manager by phone to make an inventory of problems at work from the employer representative’s perspective and discuss a time for the three-part meeting. 2) Brainstorming about solutions by both the employee and RC, and 3) the formulation of an action plan (employee and RC). In these two steps, a collaborative approach between the employee and the RC is used to brainstorm problems and solutions for RTW, prioritise them into a preliminary list of solutions, and identify the support needed to implement them. Next, 4) involves a three-part meeting between the employee, employer representative, and RC. In this meeting, the RC facilitates a dialogue between the employer representative, typically the first-line manager, and the employee and an action plan is created. In the final 5) step, the action plan's implementation, evaluation, and follow-up (employee and RC) are conducted. Further details of the intervention are provided elsewhere [8].

### Care as usual

CAU for employees with CMDs can involve medical treatment, cognitive behavioural therapy, or a combination of these. CAU can also involve the coordination of RTW. The strategies used for coordination in the control condition may include strategies for the facilitation of a dialogue between the employee and the employer to reach a consensus in the RTW. The description of coordination services is vague compared to the intervention condition, and there is variation in how coordination is delivered in different PCCs [36].

### Outcome measures

The outcomes of this analysis were categorised into self-reported sick leave, RTW, work ability, work performance, psychological symptoms, and general health.

Data were recorded using a web-based questionnaire at baseline, six months and 12 months to assess self-reported sick leave, work ability, psychological symptoms, and general health.

High-frequency data were obtained using five SMS questions sent every fourth week on 12 occasions during a 12-month study period to assess self-reported sick leave, RTW, and work performance.

Sick leave days ≤ 14 are not registered in the Social Insurance Agency’s register data; therefore, self-reported sick leave was recorded at baseline and 12-month follow-up using the categorical item “ Are you currently on sick leave?” (categorical, yes/no). In addition, sick leave was also recorded using the SMS question “How many days in the past four weeks have you been absent from work because of illness? Answer with a number between 0 and 28 days?” (continuous response 0–28). Data were recorded at baseline and every fourth week for 12 months (13 measures in total). The use of messages has demonstrated high compliance [37].

Full RTW was defined as a return to ordinary working hours for four consequtive weeks. Data were recorded using the following questions administrated by SMS: “During the last eight weeks, have you worked your ordinary working hours for an uninterrupted period of at least four weeks?” (categorical response, yes/no), and “Are you on sick leave right now? (categorical response; no, 25%, 50%, 75%, 100%) [38]. Data were recorded at baseline and every month for 12 months (13 measures in total).

Work ability was recorded at baseline, six months, and 12 months using three items from the Work Ability Index (WAI) [39], capturing work ability relating to the future (two years) and present in relation to physical as well as psychological demands.

Work performance was recorded with SMS data using two questions capturing health-related and work-environment impairment, namely: “How much did your health problems affect your performance at work? (continuous response, 0–10)”, and “How much did work environment problems affect your performance at work? (continuous response, 0–10)”[40, 41]. Data were recorded at baseline and every month for 12 months (13 measures in total).

Psychological symptoms were recorded at baseline, six-month follow-up, and 12-month follow-up using the Hospital Anxiety and Depression Scale (HAD) [42, 43] and the Self-reported Exhaustion Disorder Scale (s-ED) [44]. Self-reported quality of life/health was recorded at baseline, six-month follow-up, and 12-month follow-up using the EQ5D instrument [45] and 4 items from the Karolinska Sleep Questionnaire (KSQ) [46, 47] capturing the sleep quality dimension.

### Statistical analysis

The statistical analysis in this study involved three main parts: A cross-sectional and multivariable analysis of variance (MANOVA) of responses at baseline and six- and 12-month follow-ups; a longitudinal analysis of SMS data; and a survival analysis of self-reported RTW.

Group differences were analysed with multivariate analysis of variance (MANOVA). Differences in psychological symptoms and future work ability over time were estimated with a paired t-test and significance from a two-sided p-value, where statistical significance was set to > 0.05. The ordinal variables S-ED, future work ability, current physical work ability, and current psychological work ability were treated as count data.

The SMS data were analysed using GEE (Generalized Estimating Equations) models with different link functions depending on the outcome. The continuous outcome of days on sick leave was modelled as count data using the Poisson family and the log-link, while the remaining continuous outcomes were modelled as normal. Binomial and ordinal outcomes were modelled using the logit link. Participant ID was used as a grouping variable, and treatment and time were considered independent variables. Further, bootstrap was applied to estimate standard errors and confidence intervals for the fitted values, and models were run both with and without an interaction between treatment and time.

An interval-censored cox regression model was used for a survival analysis of self-reported RTW. Intervals were constructed between when participants fulfilled the RTW criteria and their previous data entry. In the first model, full RTW was used as an event; in the second model, the first decrease in sick leave was used as an event.

STATA 15.1 was used for the survival analysis, while R version 4.2.2 was used for the other analyses. IBM SPSS statistics, version 28.01.1, was used for the multivariate ANOVA and paired t-test [48]. The threshold for statistical significance was set at a p-value of less than 0.05.

## Results

A total of 1506 employees were eligible and invited to participate in the trial. One hundred ninety-nine (13.2%) individuals responded to the invitation to participate and received the PSI-WPI (*n* = 87) or CAU (*n* = 112). Randomization was performed on the RC level; nine delivered the PSI-WPI and 10 CAU. One participant withdrew, one was excluded as the employment ended, nine did not answer the baseline survey, and three were removed from the analysis due to missing data. The analysis included 81 subjects who received the PSI-WPI and 104 who received CAU. Baseline characteristics are presented in Table 1. More participants in the PSI-WPI group worked as managers/had a managerial position compared to CAU; other than that, the groups were similar across baseline variables.

**Table 1:** Baseline characteristics of participants

**Variables**		**PSI-WPI (*n* = 81)**	**CAU (*n* = 104)**
**Age, mean (SD)**		41.4 (11.2)	42.6 (9.3)
**Female, n (%)**		69 (85.2)	89 (85.6)
**Living with a partner, n (%)**		60 (75.0)	73 (72.0)
**Children under the age of 16 living at home, n (%)**		44 (55.7)	59 (57.3)
**Main household responsibility, n (%)**	Myself	42 (52.5)	59 (57.3)
	Someone else	2 (2.5)	7 (6.8)
	Equal share	36 (45.0)	37 (35.9)
**Education level, n (%)**	Primary / secondary education	45 (56.2)	56 (54.9)
	Higher education/university	35 (43.8)	46 (45.1)
**Works as manager, yes, n (%)**		16 (20.3)*	8 (7.8)*
**Type of work, n (%)**	Psychologically demanding	42 (53.2)	49 (47.6)
	Physically demanding	3 (3.8)	4 (3.9)
	Both mentally and physically demanding	34 (43.0)	50 (48.5)
**Employment conditions, n (%)**	Permanent position	71 (89.9)	99 (96.1)
	Temporary position	8 (10.1)	4 (3.9)
**Work experience, at current workplace, n (%)**	Less than 1 year	17 (21.5)	13 (12.6)
	1–2 years	11 (13.9)	30 (29.1)
	3–5 years	19 (24.1)	24 (23.3)
	6–10 years	13 (16.5)	13 (12.6)
	More than 10 years	19 (24.1)	23 (22.3)
**Outcomes at baseline**			
**HADS anxiety categories, n (%)**	Normal (0–7)	21 (26.6)	30 (29.4)
	Mild (8–10)	16 (20.3)	27 (26.5)
	Moderate/severe (11–21)	42 (53.2)	45 (44.1)
**HADS depression categories, n (%)**	Normal (0–7)	29 (36.7)	37 (36.3)
	Mild (8–10)	25 (31.6)	30 (29.4)
	Moderate/severe (11–21)	25 (31.6)	35 (34.4)
**s-ED, n (%)**	No SED	11 (13.9)	12 (12.0)
	Light/moderate SED	11 (13.9)	16 (16.0)
	Pronounced SED	57 (72.2)	72 (72.0)
**WAI, future, able to work in current job in 2 years, n (%)**	Probably not	16 (20.5)	14 (13.7)
	Unsure	25 (32.1)	34 (33.3)
	Yes, probably	37 (47.4)	54 (52.9)
**WAI, work ability given current physical job demand, n (%)**	Very Good	16 (20.3)	14 (13.6)
	Quite good	23 (29.1)	27 (26.2)
	Moderate	18 (22.8)	32 (31.1)
	Quite poor	16 (20.3)	20 (19.4)
	Very poor	6 (7.6)	10 (9.7)
**WAI, work ability given current psychological job demand, n (%)**	Very Good	0 (0.0)	2 (1.9)
	Quite good	12 (15.2)	12 (11.7)
	Moderate	15 (19.0)	21 (20.4)
	Quite poor	34 (43.0)	49 (47.6)
	Very poor	18 (22.8)	19 (18.4)

*PSI-WPI *Problem-solving intervention with workplace involvement, *CAU *Care as usual, *HADS *Hospital Anxiety and Depression Scale, *s-ED *Self-reported Exhaustion Disorder Scale, *WAI *Work Abillity Index

*Pearson Chi-Square, *p* = 0.014

The mean values of psychological symptoms, health, and work ability and the results from the multivariate ANOVA are reported in Table 2. The PSI-WPI had no significant effect on psychological outcomes from baseline to six months or from baseline to 12 months when compared to CAU. The only statistically significant difference was found for self-rated health (EQ5D), which was in favour of the CAU group when compared from baseline to 6-month follow-up, with a mean difference of -8.44 (-14.84, -2.04). Regarding work ability (WAI), PSI-WPI had no significant effect on either future-, physical-, or psychological work ability. Even if no differences between the groups were found, psychological symptoms and work ability improved over time for both groups (Supplementary Table 1).

**Table 2:** Mean values of psychological symptoms and workability at baseline (BL), 6 months and 12 months. Multivariate analysis of variance (MANOVA) of outcomes from baseline to 6 months and baseline to 12 months

	**Mean value (SD)**						**Mean difference (95% CI)**^**a**^			
	**BL**	**n**	**6 mo**	**n**	**12 mo**	**n**	**BL – 6 mo**		**BL – 12 mo**	**n**
**HAD—Anxiety**										
PSI-WPI	11.63 (4.47)	79	8.06 (3.87)	70	7.83 (4.47)	63	0.019 (-1.07, 1.11)		0.014 (-1.26, 1.28)	57
CAU	10.94 (4.40)	102	8.08 (4.34)	90	7.45 (4.18)	73				72
**HAD—Depression**										
PSI-WPI	9.84 (4.07)	79	6.91 (4.27)	70	6.30 (4.57)	63	0.82 (-0.34, 1.99)		-0.44 (-1.74, 0.87)	57
CAU	9.98 (4.21)	102	6.80 (4.03)	90	6.58 (4.52)	76				72
**Self-reported exhaustion, S-ED**										
PSI-WPI	1.58 (0.73)	61	0.90 (0.87)	69	0.84 (0.92)	62	0.20 (-0.14, 0.52)		0.15 (-0.18, 0.49)	45
CAU	1.60 (0.70)	82	0.88 (0.86)	88	0.76 (0.82)	72				54
**Self-rated health, EQ5D**										
PSI-WPI	42.57 (19.97)	79	60.71 (21.67)	58	61.13 (22.83)	63	**-8.44 (-14.84, -2.04)**		-3.50 (-11.63, 4.62)	45
CAU	48.13 (20.35)	100	66.18 (18.12)	74	66.50 (17.52)	74				50
**Sleep quality, KSQ**										
PSI-WPI	3.50 (1.15)	76	2.53 (1.37)	71	2.21 (1.31)	62	-0.004 (-0.43, 0.42)		-0.15 (-0.54, 0.24)	55
CAU	3.26 (1.37)	101	2.55 (1.22)	88	2.31 (1.19)	77				71
**Future work ability, WAI**										
PSI-WPI	2.27 (0.78)	78	2.46 (0.77)	71	2.45 (0.72)	62	0.01 (-0.21, 0.23)		0.08 (-0.15, 0.30)	56
CAU	2.39 (0.72)	102	2.49 (0.69)	90	2.43 (0.70)	77				72
**Current physical work ability, WAI**										
PSI-WPI	2.66 (1.23)	79	2.23 (1.11)	71	2.25 (1.28)	65	0.18 (-0.18, 0.53)		0.05 (-0.35, 0.46)	58
CAU	2.85 (1.12)	103	2.24 (1.01)	89	2.22 (1.15)	77				72
**Current psychological work ability, WAI**										
PSI-WPI	3.73 (0.98)	79	2.70 (1.08)	70	2.58 (1.24)	65	0.10 (-0.23, 0.43)		-0.09 (-0.50, 0.31)	59
CAU	3.69 (0.97)	103	2.67 (0.99)	89	2.61 (1.15)	77				73

*HAD *Hospital Anxiety and Depression Scale (0 to 21, higher score equals more symptoms), *s-ED *Self-reported Exhaustion Disorder Scale (0 to 2, higher score equals more symptoms), EQ5D self-rated health (0 to 100, higher score equals better self-rated health), *KSQ *Karolinska Sleep Questionnaire (0 to 5, lower score equals less symptoms and better sleep), *WAI *Work Ability Index (1 to 5, lower score equals better work ability)

^a^Estimeated mean over the study period

**Fig. 1:**
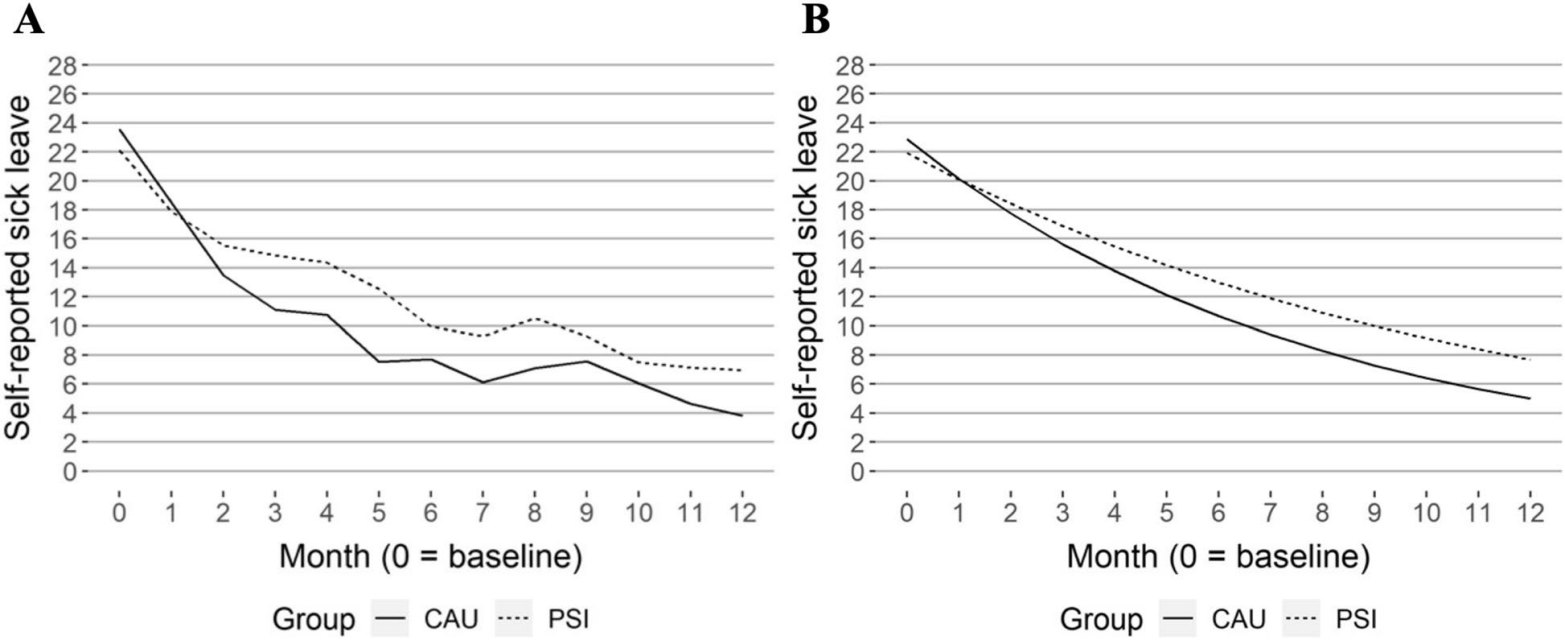
Sick leave, descriptive (**A**) and GEE estimated values (**B**)

**Fig. 2:**
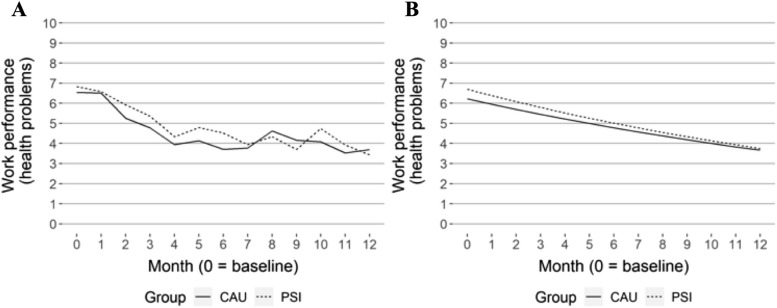
Impaired work performance related to health problems, descriptive (**A**) and GEE estimated values (**B**)

**Fig. 3:**
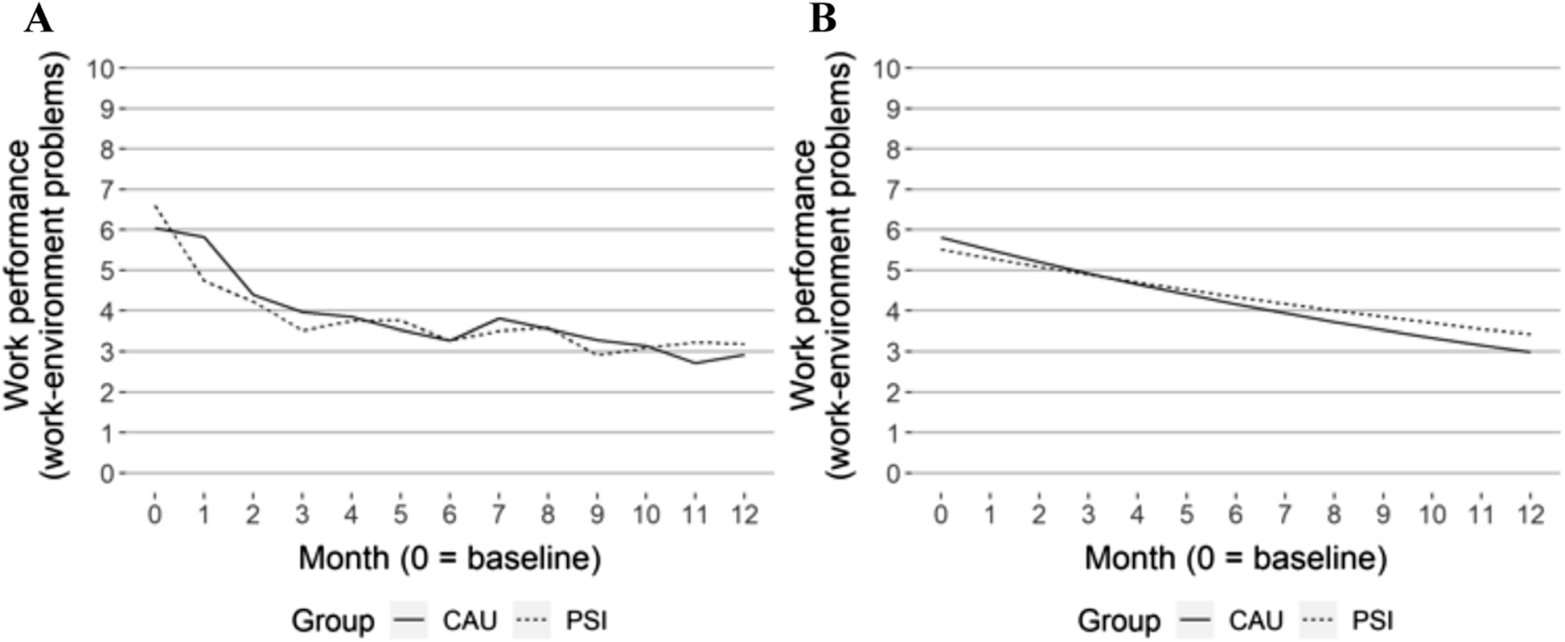
Impaired work performance related to work environment problems, descriptive (**A**) and GEE estimated values (**B**)

High-frequency data recorded by SMS on self-reported sick leave and work performance are reported in Table 3 and Figs. 1a, b, 2a, b, 3a, and b. In the longitudinal model, there was a statistically significant difference in the estimates of sick leave measured every fourth week, in favour of the CAU group, from months three to 12, see Table 3 and Fig. 1b. However, when estimated in one longitudinal model, there were no statistically significant differences between groups, see Table 4. There were no statistically significant differences in impairment of work performance between groups in the longitudinal models.

**Table 3:** Mean values and SD for each group per month for sickness leave and impaired work performance during the follow-up period. Estimated mean differences for each group per month correspond to GEE regression models. (CAU – PSI-WPI)

**Month**		**B**	**1**	**2**	**3**	**4**	**5**	**6**	**7**	**8**	**9**	**10**	**11**	**12**
**Descriptive figures**														
**Sick leave**^**C**^**, **Fig. 1a														
Mean	PSI-WPI	22.12	17.9	15.5	14.9	14.3	12.6	9.9	9.3	10.5	9.3	7.5	7.1	6.9
SD		9.6	11.7	12.9	12.7	13.3	13.5	12.6	12.5	13.0	12.6	11.8	11.5	11.3
Mean	CAU	23.6	18.5	13.5	11.1	10.7	7.5	7.7	6.1	7.1	7.6	6.0	4.6	3.8
SD		8.1	11.2	12.6	12.3	12.2	11.3	11.3	10.5	11.0	11.9	10.6	9.6	8.9
**Impaired work performance in relation to health problems**^**D**^**, **Fig. 2a														
Mean	PSI-WPI	6.8	6.6	5.9	5.4	4.3	4.8	4.5	3.9	4.3	3.7	4.7	3.9	3.4
SD		2.3	4.3	5.4	4.4	3.4	4.4	4.9	4.4	4.5	4.4	5.9	4.6	3.3
Mean	CAU	6.5	6.5	5.3	4.8	3.9	4.1	3.7	3.8	4.6	4.2	4.1	3.5	3.7
SD		2.7	4.3	3.8	3.9	3.1	4.8	3.4	4.1	4.1	5.0	4.3	3.1	3.1
**Impaired work performance in relation to work-environment problems**^**D**^**, **Fig. 3a														
Mean	PSI-WPI	6.6	4.7	4.2	3.5	3.7	3.8	3.3	3.5	3.6	2.9	3.1	3.2	3.2
SD		2.6	3.5	3.6	3.2	3.4	3.1	2.9	3.3	3.5	3.3	3.4	3.3	3.4
Mean	CAU	6.0	5.8	4.4	4.0	3.8	3.5	3.3	3.8	3.5	3.3	3.1	2.7	2.9
SD		3.1	5.1	3.2	3.0	3.1	3.0	3.0	3.1	3.0	3.0	2.9	2.9	2.9
**Estimated figures**														
**Sick leave**^**C**^**, **Fig. 1b														
Mean	PSI-WPI	21.9	20.1	18.4	16.9	15.5	14.2	13.0	11.9	10.9	10.0	9.2	8.4	7.7
Mean	CAU	22.9	20.1	17.7	15.6	13.8	12.1	10.7	9.4	8.3	7.3	6.4	5.7	5.0
Diff		0.9	0.0	-0.7	-1.3	-1.7	-2.1	-2.3	-2.5	-2.6	-2.7	-2.7	-2.7	-2.7
95% CI	LL	-2.6	-2.7	-2.9	-3.0	-3.2	-3.5	-3.7	-3.9	-4.0	-4.0	-4.0	-4.0	-4.0
	UL	3.4	1.7	0.3	-0.5	-1.1	-1.5	-1.9	-2.0	-2.0	-2.0	-2.0	-1.9	-1.8
**Impaired work performance in relation to health problems**^**D**^**, **Fig. 2b														
Mean	PSI-WPI	6.7	6.4	6.1	5.8	5.5	5.3	5.0	4.8	4.6	4.3	4.1	3.9	3.8
Mean	CAU	6.2	6.0	5.7	5.5	5.2	5.0	4.8	4.6	4.4	4.2	4.0	3.8	3.7
Diff		-0.5	-0.4	-0.4	-0.3	-0.3	-0.3	-0.2	-0.2	-0.2	-0.2	-0.1	-0.1	-0.1
95% CI	LL	-1.2	-1.1	-0.9	-0.8	-0.7	-0.6	-0.6	-0.5	-0.5	-0.5	-0.5	-0.6	-0.6
	UL	0.2	0.2	0.1	0.0	0.0	0.0	0.0	0.0	0.1	0.1	0.2	0.3	0.3
**Impaired work performance in relation to work-environment problems**^**D**^**, **Fig. 3b														
Mean	PSI-WPI	5.5	5.3	5.1	4.9	4.7	4.5	4.3	4.2	4.0	3.9	3.7	3.6	3.4
Mean	CAU	5.8	5.5	5.2	4.9	4.7	4.4	4.2	3.9	3.7	3.5	3.3	3.2	3.0
Diff		0.3	0.2	0.1	0.0	-0.1	-0.1	-0.2	-0.2	-0.3	-0.3	-0.4	-0.4	-0.4
95% CI	LL	-0.1	-0.1	-0.1	-0.1	-0.1	-0.1	-0.1	-0.2	-0.2	-0.3	-0.3	-0.4	-0.4
	UL	1.1	0.9	0.7	0.6	0.4	0.3	0.3	0.3	0.3	0.2	0.2	0.2	0.2

*B *baseline, *CAU *care as usual, *PSI *problem-solving intervention, *SD *standard deviation, *Diff *difference, *95% CI *95% confidence interval, *LL *lower limit, *UL *upper limit, C, number of days, 0–28; D, 0–10, higher is related to more impairment

**Table 4:** Estimated (GEE model) relative risk of sick leave and work performance between groups (PSI-WPI – CAU) over 12 months

Variable	Estimate (95% CI)	p
Days on sick leave, interaction group x time (ratio of rate ratios)	1.04 (0.99, 1.09)	0.12
Impaired work performance associated with health problems, interaction group x time	-0.03 (-0.15, 0.09)	0.600
Impaired work performance associated with work-environment problems, interaction group x time	0.06 (-0.03, 0.15)	0.220

*CI *confidence interval, p, p-value

The longitudinal GEE analysis of the high-frequency SMS data on sick leave did not show a difference between groups, neither at baseline nor in how they change over time, concerning the risk of being on sick leave. Longitudinal models for high-frequency SMS data on work performance showed similar results with no difference in means between the groups at baseline or development over the 12-month period, see Table 4.

**Fig. 4:**
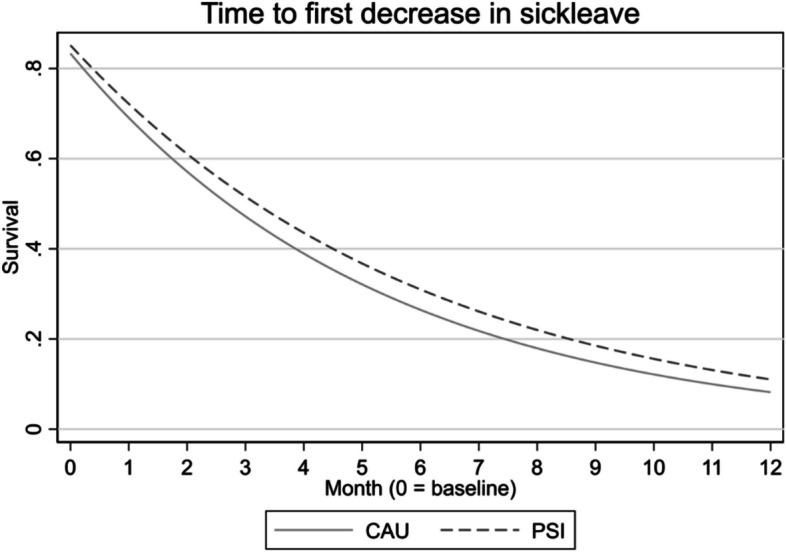
Partial return to work, survival analysis

**Fig. 5:**
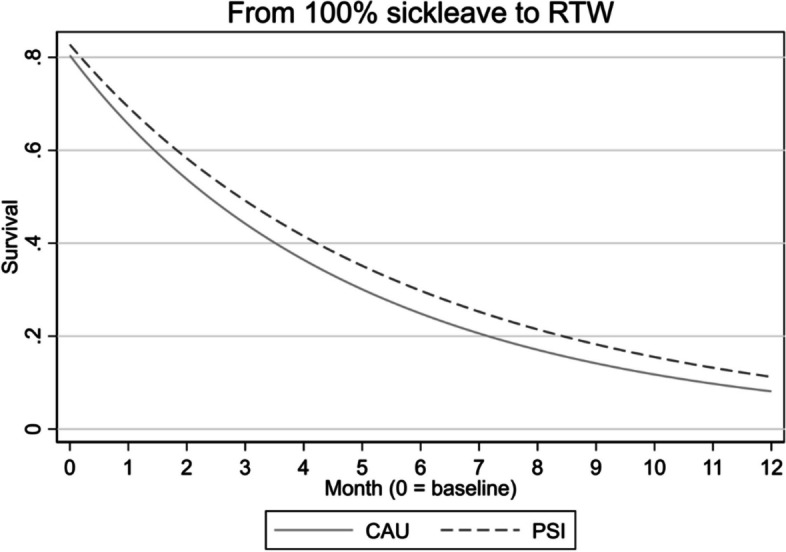
Full return to work (RTW), survival analysis

RTW was analysed in an interval-censored survival model estimating two scenarios: partial RTW and full return to ordinary working hours. A partial RTW was defined as any reduced level of sick leave from any level of sick leave, whereas full RTW was defined as the return to ordinary working hours for at least four weeks. There were no statistically significant differences in RTW between groups in either of the analyses; see Table 5 and Figs. 4 and 5.

**Table 5:** Return to Work (RTW), interval censored survival model, to account for the uncertainty in the exact return time. The parameter of interest is group allocation

Interval	Haz. Ratio (95% CI)	p
Partial RTW	0.89 (0.65, 1.22)	0.467
Full RTW	0.87 (0.45, 1.66)	0.674

*CI *confidence interval, p, p-value

## Discussion

This study investigated if, in a primary care context, a PSI-WPI intervention added to CAU effectively reduces self-reported sick leave and psychological symptoms and improves work ability, work performance, and health compared to CAU only. No significant differences were found when analysing differences between the groups in psychological symptoms (except for self-rated health, which favoured the CAU group), self-reported sick leave, and impaired work performance. Further, no significant effect from PSI-WPI was found regarding perceived psychological-, physical- or future work ability. Finally, receiving PSI-WPI made no significant difference for a partial- or full RTW during the first year of follow-up. In conclusion, PSI-WPI resulted in no additional benefit compared to CAU for employees who, at inclusion, were sick-listed due to CMDs.

These results, which show no additional benefit of PSI-WPI, reflect the results of a similar trial of a problem-solving intervention in an occupational healthcare setting in Sweden [33]. In the study by Keus van de Poll [33], no significant differences were found for self-reported sick leave days and psychological symptoms compared to CAU. Our results are also in line with a systematic review evaluating the combination of clinical interventions in combination with workplace interventions, which showed no effect on work functioning at up to one year follow-up [25].

We anticipated that distal effects on symptoms and health outcomes would be evident, given the promising results from previous studies on RTW and the decrease in sick leave when conducted in the occupational healthcare sector [27, 33]. This anticipation was built on, for example, a Cochrane review that assigned the positive results for sick leave days reported from PSI-WPI to the integration of a clinical- and workplace intervention in improving work outcomes, but this does not seem to be sufficient to also result in positive effects on other outcomes [25]. Another Cochrane review described that problem-solving may help employees partially RTW and also found that employees who received this support returned to work 17 days earlier, although it made no difference for full RTW or distress at the 12-month follow-up [26]. Further, previous systematic reviews on work-directed problem-solving interventions with workplace involvement have mainly shown no significant improvement in psychological symptoms, and some studies show an effect when the intervention is more comprehensive and focused beyond the work situation [25]. Studies evaluating the mechanisms that affect sick leave and other outcomes are scarce in the literature today.

The current study was conducted in primary care in an attempt to see if the positive results reported from occupational health services [24] could be transferred to this context. In addition, the employees included in our trial had been on sick leave for 2–12 weeks, while the trial by Arends et al., reporting positive results, had shorter inclusion criteria and included individuals who had returned to work or were expected to return soon [24]. Moreover, in the study by Arends et al., occupational physicians were responsible for prescribing sick leave and also provided the intervention. This may have been a crucial factor in the process. In hindsight, it may be necessary to reconsider the choice of context. For example, primary care has limited access to workplaces compared to occupational health services and thus has less influence over the employer who is legally responsible for the work environment [49]. Hence, a further exploration at which point during the sick leave period an employee is susceptible to acquiring the problem-solving skills and multi-stakeholder concept is needed, something also discussed in a systematic review evaluating problem-solving interventions [50]. Reflecting on the intervention context and inclusion criteria is therefore relevant.

The structured design of the intervention, its focus on supporting the individual to identify problems and formulate solutions, and its inclusion of the first-line manager were expected to improve outcomes compared to CAU. Likewise, in a qualitative study evaluating facilitators of and barriers to participating in PSI-WPI, the structure, problem-solving process, and early involvement of the manager were reported as facilitators, while an increased time and increased number of meetings were reported as barriers [51]. Even if both groups reported better psychological outcomes and work ability over time (in a potential regression towards the mean) no additional effect from PSI-WPI was visible. This may be due to a lack of contrast between PSI-WPI and CAU or being too limited to demonstrate any clinically significant effect. Another explanation could be that there is a lack of heterogeneity in the CAU intervention, and that the variation of interventions is too great, resulting in large within-group variation. In addition, the lack of a higher RTW in PSI-WPI may be the reason why neither of the secondary outcomes demonstrates beneficial effects from the PSI-WPI. A process evaluation of the PSI-WPI is currently being conducted, which will evaluate fidelity, reach, dose delivered, and dose received and if any of these factors may have had an effect on the number of sick leave days.

We conclude that the PSI-WPI did not demonstrate any additional effect on any of our outcomes compared to CAU. A previous trial established that many employees who have returned to work still struggle with symptoms of depression and anxiety, as well as impaired work performance one year after returning to work [34]. This may be one reason why no difference between the groups was seen. In addition, studies evaluating RTW may need to re-evaluate what clinical relevance is when analysing outcomes such as sick leave days or psychological outcomes. If the clinical relevance is set to the advantage of the employee or the advantage of the healthcare or workplace, the necessary improvement may be different. A systematic review discussed the relevance for different stakeholders and the fact that the clinical relevance may differ from study to study, usually depending on the length of follow-up [25].

The trial was conducted as a cluster RCT, which is the goal standard for evaluating interventions. Several outcomes were included in the study to enable an evaluation of outcomes beyond sick leave that may result from receiving a PSI-WPI. Despite a two-year-long inclusion period (February 2018 to February 2020), the trial did not reach its planned sample size (197 were included instead of the 220 employees aimed for). Thus, the study faced somewhat limited power. In addition, there was a loss to follow-up on the different outcomes, depending on how many participants answered the surveys and text messages. There is a higher risk of type II error in underpowered studies, which means that we may have failed to establish an effect due to the small sample size.

## Conclusion

A PSI-WPI added to CAU in a primary care context, targeting employees on sick leave due to CMDs, demonstrated no additional effect on psychological symptoms, health, work ability, and self-reported sick leave compared to CAU only. Further research is needed to understand why problem-solving interventions appear to have an effect on sick leave in occupational healthcare when compared to CAU, but no effect has been established in primary care. Additionally, contextual factors, implementation factors, and other mechanisms, such as the implementation process, participants’ responses, and interactions potentially affecting sick leave, should be explored.

## Supplementary Information

Supplementary Material 1

Supplementary Material 2

## Data Availability

Due to the Swedish Ethical Review Regulation, the datasets collected and analysed for the current study are not publicly available. However, data are available upon reasonable request. Inquiries for data access should be sent to Karolinska Institutet, Institute of Environmental Medicine, Unit of Intervention and Implementation Research for Worker Health, Box 210, 171 77 Stockholm or sought by contacting the principal investigator (PI) Elisabeth Björk Brämberg, elisabeth.bjork.bramberg@ki.se. The PI will then contact the Swedish Ethical Review Authority for permission to openly share the data.

## Abbreviations

CMD: Common mental disorders
RTW: Return to Work
PSI-WPI: Problem-Solving Intervention with Workplace Involvement
PROSA: Problem-solving intervention in primary care.
CAU: Care as Usual
PCC: Primary Care Centers
RC: Rehabilitation Coordinator
HAD: Hospital Anxiety and Depression Scale
s-ED: Self-reported Exhaustion Disorder Scale
KSQ: Karolinska Sleep Questionnaire
WAI: Work Ability Index
