# Supplementary material for: The effectiveness of a problem-solving intervention with workplace involvement on self-reported sick leave, psychological symptoms and work ability: a cluster randomised clinical trial
Source: BMC Public Health. 2024 Nov 5;24:3052. doi: 10.1186/s12889-024-20564-z (PMC11536685; doi:10.1186/s12889-024-20564-z)
Supplement: Supplementary file 1 — Supplementary Material 1 [file 12889_2024_20564_MOESM1_ESM.docx]

 Extension of CONSORT for abstracts^11 12^ to reports of cluster randomised trials

| Item | Standard checklist item | | Extension for cluster trials | | | Information found: | |
| --- | --- | --- | --- | --- | --- | --- | --- |
| Title | Identification of study as randomised | | Identification of study as cluster randomised | | | Yes | |
| Trial design | Description of the trial design (for example, parallel, cluster, non-inferiority) | |  | | | Yes | |
| Methods: |  | |  | | |  | |
| Participants | Eligibility criteria for participants and the settings where the data were collected | | Eligibility criteria for clusters | | | Yes, for employees | |
| Interventions | Interventions intended for each group | |  | | | Yes | |
| Objective | Specific objective or hypothesis | | Whether objective or hypothesis pertains to the cluster level, the individual participant level, or both | | | Yes | |
| Outcome | Clearly defined primary outcome for this report | | Whether the primary outcome pertains to the cluster level, the individual participant level or both | | | Yes | |
| Randomisation | How participants were allocated to interventions | | How clusters were allocated to interventions | | | Yes | |
| Blinding (masking) | Whether or not participants, care givers, and those assessing the outcomes were blinded to group assignment | |  | | | Yes | |
| Results: |  | |  | | |  | |
| Numbers randomised | Number of participants randomised to each group | | Number of clusters randomised to each group | | | Yes | |
| Recruitment | Trial status* | |  | | |  | |
| Numbers analysed | Number of participants analysed in each group | | Number of clusters analysed in each group | | | Yes, for employees | |
| Outcome | For the primary outcome, a result for each group and the estimated effect size and its precision | | Results at the cluster or individual level as applicable for each primary outcome | | | Yes, for each group | |
| Harms | Important adverse events or side effects | |  | | | No harms to report | |
| Conclusions | General interpretation of the results | |  | | | Yes | |
| Trial registration | Registration number and name of trial register | |  | | | Yes | |
| Funding | Source of funding | |  | | | Not in abstract, but included in the manuscript | |
|  | |  | |  |  | |  |

 CONSORT 2010 checklist of information to include when reporting a cluster randomised trial

| Section/topic and item No | | | Standard checklist item | Extension for cluster designs | Page No* |
| --- | --- | --- | --- | --- | --- |
| **Title and abstract** | | | | |  |
| 1a | | | Identification as a randomised trial in the title | Identification as a cluster randomised trial in the title | 1 |
| 1b | | | Structured summary of trial design, methods, results, and conclusions (for specific guidance see CONSORT for abstracts)^11 12^ | See table 2 | 1-2 |
| **Introduction** | | | | |  |
| Background and objectives: | | |  |  |  |
| 2a | | | Scientific background and explanation of rationale | Rationale for using a cluster design | 5 |
| 2b | | | Specific objectives or hypotheses | Whether objectives pertain to the cluster level, the individual participant level, or both | 5 |
| **Methods** | | | | |  |
| Trial design: | | |  |  |  |
| 3a | | | Description of trial design (such as parallel, factorial) including allocation ratio | Definition of cluster and description of how the design features apply to the clusters | 5-6 |
| 3b | | | Important changes to methods after trial commencement (such as eligibility criteria), with reasons |  | NA |
| Participants: | | |  |  |  |
| 4a | | | Eligibility criteria for participants | Eligibility criteria for clusters | 6 |
| 4b | | | Settings and locations where the data were collected |  | 6 |
| Interventions: | | |  |  |  |
| 5 | | | The interventions for each group with sufficient details to allow replication, including how and when they were actually administered | Whether interventions pertain to the cluster level, the individual participant level, or both | 6-7 |
| Outcomes: | | |  |  |  |
| 6a | | | Completely defined prespecified primary and secondary outcome measures, including how and when they were assessed | Whether outcome measures pertain to the cluster level, the individual participant level, or both | 7-8 |
| 6b | | | Any changes to trial outcomes after the trial commenced, with reasons |  | NA |
| Sample size: | | |  |  |  |
| 7a | | | How sample size was determined | Method of calculation, number of clusters(s) (and whether equal or unequal cluster sizes are assumed), cluster size, a coefficient of intracluster correlation (ICC or *k*), and an indication of its uncertainty | NA |
| 7b | | | When applicable, explanation of any interim analyses and stopping guidelines |  | NA |
| **Randomisation** | | | | |  |
| Sequence generation: | | |  |  |  |
| 8a | | | Method used to generate the random allocation sequence |  | 6 |
| 8b | | | Type of randomisation; details of any restriction (such as blocking and block size) | Details of stratification or matching if used | NA |
| Allocation concealment mechanism: | | |  |  |  |
| 9 | | | Mechanism used to implement the random allocation sequence (such as sequentially numbered containers), describing any steps taken to conceal the sequence until interventions were assigned | Specification that allocation was based on clusters rather than individuals and whether allocation concealment (if any) was at the cluster level, the individual participant level, or both | 6 |
| Implementation: | | |  |  |  |
| 10a | | |  | Who generated the random allocation sequence, who enrolled clusters, and who assigned clusters to interventions | 6 |
| 10b | | |  | Mechanism by which individual participants were included in clusters for the purposes of the trial (such as complete enumeration, random sampling) | 6 |
| 10c | | |  | From whom consent was sought (representatives of the cluster, or individual cluster members, or both) and whether consent was sought before or after randomisation | 19 |
| Blinding: | | |  |  |  |
| 11a | | | If done, who was blinded after assignment to interventions (for example, participants, care providers, those assessing outcomes) and how |  | 6 |
| 11b | | | If relevant, description of the similarity of interventions |  | NA |
| Statistical methods: | | |  |  |  |
| 12a | | | Statistical methods used to compare groups for primary and secondary outcomes | How clustering was taken into account | 9 |
| 12b | | | Methods for additional analyses, such as subgroup analyses and adjusted analyses |  | NA |
| **Results** | | | | |  |
| Participant flow (a diagram is strongly recommended): | | |  |  | NA |
| 13a | | | For each group, the numbers of participants who were randomly assigned, received intended treatment, and were analysed for the primary outcome | For each group, the numbers of clusters that were randomly assigned, received intended treatment, and were analysed for the primary outcome | 10-11 |
| 13b | | | For each group, losses and exclusions after randomisation, together with reasons | For each group, losses and exclusions for both clusters and individual cluster members | NA |
| Recruitment: | | |  |  |  |
| 14a | | | Dates defining the periods of recruitment and follow-up |  | 6 |
| 14b | | | Why the trial ended or was stopped |  | NA |
| Baseline data: | | |  |  |  |
| 15 | | | A table showing baseline demographic and clinical characteristics for each group | Baseline characteristics for the individual and cluster levels as applicable for each group | 10-11 |
| Numbers analysed: | | |  |  |  |
| 16 | | | For each group, number of participants (denominator) included in each analysis and whether the analysis was by original assigned groups | For each group, number of clusters included in each analysis | 10-13 |
| Outcomes and estimation: | | |  |  |  |
| 17a | | | For each primary and secondary outcome, results for each group, and the estimated effect size and its precision (such as 95% confidence interval) | Results at the individual or cluster level as applicable and a coefficient of intracluster correlation (ICC or *k*) for each primary outcome | 11-15 |
| 17b | | | For binary outcomes, presentation of both absolute and relative effect sizes is recommended |  | 11-15 |
| Ancillary analyses: | | |  |  |  |
| 18 | | | Results of any other analyses performed, including subgroup analyses and adjusted analyses, distinguishing prespecified from exploratory |  | 11-15 |
| Harms: | | |  |  |  |
| 19 | | | All important harms or unintended effects in each group (for specific guidance see CONSORT for harms106) |  | NA |
| **Discussion** | | | | |  |
| Limitations: | | |  |  |  |
| 20 | | | Trial limitations, addressing sources of potential bias, imprecision, and, if relevant, multiplicity of analyses |  | 16-18 |
| Generalisability: | | |  |  |  |
| 21 | | | Generalisability (external validity, applicability) of the trial findings | Generalisability to clusters and/or individual participants (as relevant) | 16-18 |
| Interpretation: | | |  |  |  |
| 22 | | | Interpretation consistent with results, balancing benefits and harms, and considering other relevant evidence |  | 16-18 |
| **Other information** | | | | |  |
| Registration: | | |  |  |  |
| 23 | | | Registration number and name of trial registry |  | 3 |
| Protocol: | | |  |  |  |
| 24 | | | Where the full trial protocol can be accessed, if available |  | 6 |
| Funding: | | |  |  |  |
| 25 | | | Sources of funding and other support (such as supply of drugs), role of funders |  | 20 |
|  |  |  |  |  |  |
