# Supplementary material for: The effectiveness of a problem-solving intervention with workplace involvement on self-reported sick leave, psychological symptoms and work ability: a cluster randomised clinical trial
Source: BMC Public Health. 2024 Nov 5;24:3052. doi: 10.1186/s12889-024-20564-z (PMC11536685; doi:10.1186/s12889-024-20564-z)
Supplement: Supplementary file 2 — Supplementary Material 2 [file 12889_2024_20564_MOESM2_ESM.docx]

Supplementary Table 1. Paired t-test, baseline to 6 months and baseline to 12 months for PSI-WPI and CAU

|  | | **Baseline to 6 months** | | | **Baseline to 12 months** | | |
| --- | --- | --- | --- | --- | --- | --- | --- |
| **Variable** | **Group** | **Mean difference** | **95% CI** | **p** | **Mean difference** | **95% CI** | **p** |
| HAD anxiety | PSI-WPI | 3.39 | 2.54;4.25 | <0.001 | 3.60 | 2.51;4.69 | <0.001 |
|  | CAU | 2.94 | 2.19;3.67 | <0.001 | 3.46 | 2.60;4.32 | <0.001 |
| HAD depression | PSI-WPI | 2.78 | 1.87;3.67 | <0.001 | 3.79 | 2.76;4.83 | <0.001 |
|  | CAU | 3.51 | 2.73;4.29 | <0.001 | 3.46 | 2.53;4.39 | <0.001 |
| Self-reported exhaustion | PSI-WPI | 0.71 | 0.44;0.99 | <0.001 | 0.75 | 0.49;1.02 | <0.001 |
|  | CAU | 0.79 | 0.57;1.00 | <0.001 | 0.91 | 0.69;1.14 | <0.001 |
| Self-rated health | PSI-WPI | -18,14 | -23,31;-12,97 | <0.001 | -21.14 | -27.89;-14.39 | <0.001 |
|  | CAU | -19.60 | -24.21;-14.98 | <0.001 | -19.19 | -25.98;-12.39 | <0.001 |
| Sleep quality | PSI-WPI | 0.94 | 0.57;1.30 | <0.001 | 1.28 | 0.93;1.64 | <0.001 |
|  | CAU | 0.74 | 0.47;1.00 | <0.001 | 1.04 | 0.75;1.32 | <0.001 |
| Future work ability, WAI | PSI-WPI | -0.25 | -0.46;-0.31 | <0.026 | -0.13 | -0.36;0.10 | 0.252 |
|  | CAU | -0.09 | -0.22;0.04 | 0.171 | 0.00 | -0.15;0.15 | 1.000 |
| Current phys. work ability | PSI-WPI | 0.46 | 0.14;0.79 | 0,006 | 0.41 | 0.02;0.81 | 0.040 |
|  | CAU | 0.64 | 0.40;0.88 | <0.001 | 0.54 | 0.20;0.88 | 0.003 |
| Current psych. work ability | PSI-WPI | 1.06 | 0.78;1.34 | <0.001 | 1.28 | 0.96;1.60 | <0.001 |
|  | CAU | 1.02 | 0.79;1.26 | <0.001 | 1.08 | 0.76;1.40 | <0.001 |

**Paired samples t-test, significance from two-sided p-value.*
